# The Impact of Chemosensitivity on the Outcome of Brain Metastases in Small-Cell Lung Cancer: A Retrospective Analysis

**DOI:** 10.3390/curroncol29100631

**Published:** 2022-10-21

**Authors:** Jintao Ma, Chunliu Meng, Jia Tian, Kai Ren, Huijun Jia, Meng Yan, Liming Xu, Lujun Zhao

**Affiliations:** Key Laboratory of Cancer Prevention and Therapy, Department of Radiation Oncology, Tianjin Medical University Cancer Institute and Hospital, National Clinical Research Center for Cancer, Tianjin’s Clinical Research Center for Cancer, Tianjin 300060, China

**Keywords:** small-cell lung cancer, progression, brain metastases, chemosensitivity

## Abstract

Purpose: The purpose of this study was to investigate the prognostic differences between patients with small-cell lung cancer (SCLC) with different chemosensitivity to first-line chemotherapy who developed brain metastasis (BM) as the first site of progression. Methods: Patients with a BM after first-line treatment in the Tianjin Cancer Hospital were retrospectively analyzed. According to the time-free interval (TFI) between the completion of first-line chemotherapy and the onset of the BM, the patients were divided into the chemo-sensitive group (TFI ≥ 90 days, *n* = 145) and the chemo-resistant group (TFI < 90 days, *n* = 97). The survival time, which was calculated from the diagnosis of the BM, was analyzed after the onset of brain metastasis (BM-OS). Survival curves were plotted using the Kaplan–Meier method, and differences between groups were compared using the log-rank test. Results: In total, the median BM-OS was 8.4 months. The median BM-OS in the chemo-sensitive group was 8.8 months, and it was 8.0 months in the chemo-resistant group (*p* = 0.538). In patients without extracranial progression (*n* = 193), the median BM-OSes in the chemo-sensitive and chemo-resistant groups were 9.4 months and 9.7 months, respectively (*p* = 0.947). In patients with extracranial progression (*n* = 49), the median BM-OSes in the chemo-sensitive and chemo-resistant groups were 5.4 months and 4.2 months, respectively (*p* = 0.161). **Conclusions**: After the development of a BM as the first site of progression following chemotherapy in patients with SCLC, the prognosis of chemo-sensitive patients was not necessarily superior to chemo-resistant patients, especially in patients without extracranial progression.

## 1. Introduction

Small-cell lung cancer (SCLC) is highly aggressive and about two-thirds of cases are an extensive disease (ED) at the time of diagnosis [1]. The brain is a common site of distant metastasis, accounting for approximately 18% of cases at initial diagnosis, and can reach 50–65% of cases within two years [2,3]. It was previously believed that systemic chemotherapy played a limited role in treating intracranial lesions because of the difficulty in passing the intact blood–brain barrier (BBB). However, several research studies have suggested that the BBB may not be the factor impeding the successful treatment of brain metastases (BMs) with chemotherapy agents, and the observed objective response rate has ranged from 27–82% [4,5]. However, most of these were outdated studies with a limited number of samples; therefore, the effect of chemotherapy agents on the BMs of patients with SCLC is still unclear.

Although SCLC responds well to chemoradiotherapy, approximately 50% of patients relapse within one year. Patients with recurrent SCLC who progress after first-line chemotherapy are traditionally classified into chemo-sensitive cases (time-free interval (TFI) ≥ 90 days) and chemo-resistant cases (TFI < 90 days). Previous studies have found that there was difference in the survival outcome and efficacy of second-line treatment between sensitive relapse and resistant relapse, and the former’s patients had better prognoses [6,7,8].

Radiation therapy is the standard of care in patients with SCLC who develop a BM [1,9]. However, whether the prognosis is related to chemosensitivity in patients with a BM is unknown. This retrospective study analyzes the differences in prognosis between patients with SCLC with different chemosensitivity to first-line chemotherapy who developed BMs as the first site of progression.

## 2. Materials and Methods

Consecutive cases admitted to the Tianjin Cancer Hospital from January 2012 to October 2020 were retrospectively analyzed (bc2022166). The use of samples and data involved in the study was approved by the Institutional Review Board of Tianjin Medical University Cancer Institute and Hospital. Informed consent for the scientific usage of clinical data was obtained from all patients. Inclusion criteria were as follows: (1) the diagnosis of SCLC was confirmed by histopathology or cytology; (2) brain metastases were documented by pathology or imaging (magnetic resonance imaging with contrast or computed tomographic with contrast), with or without neurological symptoms; (3) the first site of initial treatment failure was the brain; (4) the number of first-line platinum-doublet chemotherapy before BM was greater than two cycles. The definition of chemo-sensitive status was a TFI ≥ 90 days, and chemo-resistant status was defined as a TFI < 90 days. 

The endpoints were survival time after the onset of brain metastasis (BM-OS) and overall survival (OS). The onset of brain metastasis was calculated from the date of diagnosis of the BMs to the date of death from any cause, and OS was calculated from the date of diagnosis of SCLC to the date of death due to any cause. The last follow-up was on 13 May 2022. The Kaplan–Meier survival analysis evaluated median BM-OS and OS, and survival differences between groups were compared using the log-rank test. Comparison between the categorical variables was analyzed using the Chi-squared test or Fisher’s exact test. All statistical tests were bilateral, and *p* < 0.05 was considered significant. Statistical analyses were undertaken using SPSS 26.0 software (IBM, Chicago, IL, USA).

## 3. Results

**Figure 1:**
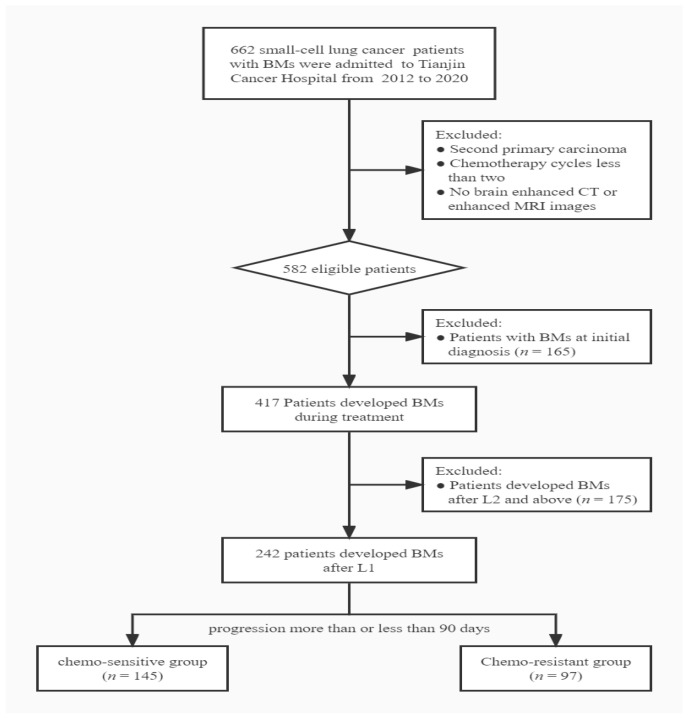
Patients’ selection diagram. BM: brain metastasis; L1: first-line chemotherapy; L2: second-line chemotherapy.

Figure 1 illustrates the inclusion and exclusion criteria for the study. According to the criteria, 242 eligible patients were enrolled with a median follow-up of 49.3 months. Among them, 145 patients were chemo-sensitive, and 97 patients were chemo-resistant. According to the Veterans Administration Lung Study Group definition of limited disease (LD) and ED, 155 patients had LD and 87 had ED at initial diagnosis. One hundred and ninety-three patients had intracranial progression alone, and forty-nine had concurrent extracranial progression.

All patients received at least two cycles of etoposide-platinum regimens before BM. For LD-SCLC patients at initial diagnosis, 6 patients (3.9%) received surgery, and adjuvant chemotherapy was undertaken with or without thoracic radiation therapy (TRT); 63 patients (40.6%) received concurrent chemoradiotherapy; 76 patients (49.0%) received sequential chemoradiotherapy; and 10 patients (6.5%) received chemotherapy alone. For ED-SCLC patients at initial diagnosis, 27 patients (31.0%) underwent chemotherapy alone, 60 patients (69.0%) underwent TRT, and 10 patients (11.5%) received chemotherapy combined with immunotherapy. At the same time, an evaluation was performed for patients who underwent surgery after the completion of postoperative adjuvant therapy. After completion of primary therapy, surveillance was performed every 3 months during the first two years, then every 6 months during the third year, and annually after that. 

After the diagnose of BMs, 109 patients (45.0%) received local therapy, 27 patients (11.2%) received systemic therapy, 86 patients (35.5%) received the combination of local and systemic therapy, and 20 patients (8.3%) only received supportive care. Among the patients who received local treatment, 142 (72.8%) were treated with whole brain radiation (WBRT), 44 (22.6%) were treated with WBRT plus a radiation boost, 4 (2.1%) were treated with stereotactic radiation therapy (SRT), and 5 (2.5%) were treated with surgical resection. 

The characteristics of patients between the chemo-sensitive group and the chemo-resistant group are provided in Table 1. In total, 242 patients had a median age of 61 years (range 29–78 years), and 78.1% were male. More chemo-sensitive patients received TRT (87.6%) and prophylactic cranial irradiation (PCI) (10.3%) during first-line treatment, and the other baseline characteristics were similar between the two groups. The majority of patients (83.5%) received brain radiation after brain metastasis (Table 1).

**Figure 2:**
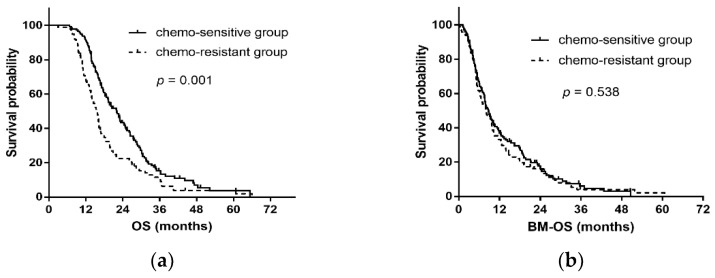
Kaplan–Meier curves for overall survival and survival time after BM in all patients between two groups. (**a**) Overall survival; (**b**) Survival time after BM. BM: brain metastasis.

At the end of the last follow-up, 212 patients (87.6%) died, and 30 (12.4%) survived. For all patients, the median OS was 18.2 months, with 1- and 3-year OS rates of 81.7% and 13.4%, respectively, and the median BM-OS was 8.4 months, with 1- and 3-year OS rates of 35.3% and 5.2%, respectively. The median OS times in chemo-sensitive and chemo-resistant patients were 22.0 months and 15.6 months, respectively (*p* = 0.001). The median BM-OS times were 8.8 months and 8.0 months between the chemo-sensitive and chemo-resistant groups, respectively (*p* = 0.538) (Figure 2).

In LD-SCLC patients (*n* = 155), the median OS was 21.8 months, and the median BM-OS was 10.5 months. Between the chemo-sensitive group and the chemo-resistant group, the median OS times were 24.8 months and 17.9 months, respectively (*p* = 0.01), and the median BM-OS times were 10.4 months and 11.4 months, respectively (*p* = 0.867). In ED-SCLC patients (*n* = 87), the median OS was 15.2 months, and the median BM-OS was 5.7 months. The median OS was 16.8 months in the chemo-sensitive group versus 12.8 months in the chemo-resistant group (*p* = 0.002). The median BM-OS times were 5.8 months and 5.3 months, respectively, in the two groups (*p* = 0.451).

**Figure 3:**
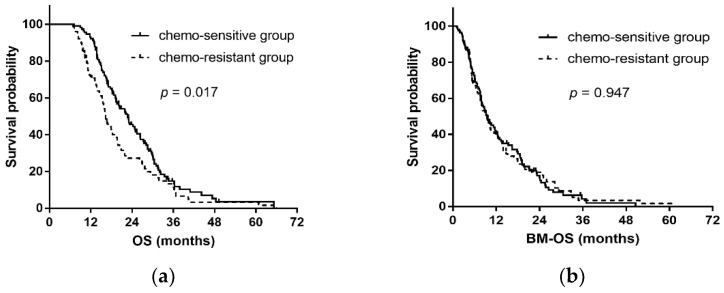
Kaplan–Meier curves for overall survival and survival time after BM in patients without extracranial progression. (**a**) Overall survival; (**b**) Survival time after BM. BM: brain metastasis.

**Figure 4:**
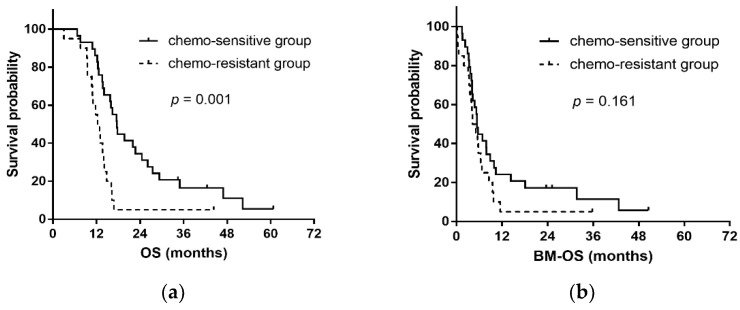
Kaplan–Meier curves for overall survival and survival time after BM in patients with extracranial progression. (**a**) Overall survival; (**b**) Survival time after BM. BM: brain metastasis.

In patients without extracranial progression, the median OS times were 22.7 months and 16.3 months in the chemo-sensitive group and the chemo-resistant group, respectively (*p* = 0.017), and the median BM-OS times were 9.4 months and 9.7 months, respectively (*p* = 0.947) (Figure 3). In patients with extracranial progression (*n* = 49), the median OS times were 17.6 months and 12.3 months in the chemo-sensitive group and the chemo-resistant group, respectively (*p* = 0.002), and the median BM-OS times were 5.4 months and 4.2 months, respectively (*p* = 0.161) (Figure 4).

## 4. Discussion

This retrospective study observed that chemo-sensitive patients had longer OS than chemo-resistant patients regardless of the initial stage at diagnosis. However, after the development of a BM, the differences in the BM-OS between the two groups were no longer significant, especially in patients without extracranial progression. Therefore, the chemosensitivity status may have limited applicability to predicting the response to second-line treatment and prognosis in BMs. 

The treatment of progressed SCLC is a challenge, especially in those who are resistant to first-line chemotherapy, because of the lack of effective second-line treatment [10,11,12,13]. Previous studies have suggested that chemosensitivity is an independent risk factor and is associated with survival time and the response to second-line therapy in patients with relapsed SCLC. Much research has been carried out in the modern era on whether chemotherapy sensitivity is related to prognosis, and researchers have confirmed the prognostic value of chemotherapy sensitivity status for relapsed SCLC [14,15]. 

However, the brain is a special site of progression, so the choice of local or systemic therapy as the primary treatment has not yet been determined [16,17,18,19]. Whole brain radiation therapy is now the standard treatment in many guidelines [1,9]. For patients with a limited number of BMs, an additional radiation boost to WBRT or SRT can be recommended [16,17]. Several studies have suggested that the occurrence of BMs is a sign of systemic failure of tumor control; therefore, the treatment of BMs should focus on chemoradiotherapy [20,21,22]. A respective study in 2021 observed that the combination of WBRT and etoposide-platinum agents could prolong OS in SCLC patients with BMs [21]. However, another prospective trial did not obtain similar results [23]. At present, the efficacy of chemotherapy for BMs has not been fully clarified.

Whether the prognosis of patients who developed BMs after first-line treatment is related to chemosensitivity is worthy of further analysis. This study did not find that the prognosis after BMs was associated with chemosensitivity. The further stratified analysis demonstrated that the median BM-OS also failed to reach a statistical difference between the chemo-sensitive and chemo-resistant groups in patients without extracranial progression. The reason why chemo-sensitive patients had a longer progression-free survival but a similar BM-OS to chemo-resistant patients is possibly due to the majority of patients in the two groups receiving brain radiation therapy. However, in patients with extracranial progression, the median BM-OS tended to benefit from being sensitive to first-line treatment. This may be due to the better control for extracranial lesions with second-line chemotherapy in chemo-sensitive patients than in chemo-resistant patients. 

There are several limitations in the analysis. First, this is a respective study with selection bias limitations, and the conclusions should be validated in further prospective studies. Second, the proportion of patients with extracranial progression was relatively small, and the results demonstrated a trend, but failed to reach a statistical significance. Third, some cases in the study received immunotherapy in the first-line treatment, and the effect of chemoimmunotherapy on the prognosis for SCLC patients with BMs requires further study to confirm.

## 5. Conclusions

It is believed that this is the first study to investigate the association between prognosis and chemosensitivity status in patients with SCLC who developed BM as the first site of progression after chemotherapy. This study observed that, after the development of BMs, there was no significant difference between the chemo-sensitive group and the chemo-resistant group, especially in the subset of patients without extracranial progression. This study’s findings are noteworthy and should be considered for confirmation by prospective clinical studies.

## Figures and Tables

**Table 1:** Patient characteristics by sensitivity to first-line treatment.

Characteristics	Resistant Group *n* (%)	Sensitive Group *n* (%)	*p* Value
Gender			0.693
male	77 (79.4)	112 (77.2)	
female	20 (20.6)	33 (22.8)	
Age/year			00.755
<65	72 (74.2)	105 (72.4)	
≥65	25 (25.8)	40 (27.6)	
KPS score			0.477
<80	8 (8.2)	16 (11.0)	
≥80	89 (91.8)	129 (89.0)	
Smoke			0.235
yes	81 (83.5)	112 (77.2)	
no	16 (16.5)	33 (22.8)	
Disease extent at initial diagnosis			0.561
LD	60 (61.9)	95 (65.5)	
ED	37 (38.1)	50 (34.5)	
Initial treatment modality			0.003
chemotherapy	22 (22.7)	18 (12.4)	
sequential chemoradiotherapy	36 (37.1)	86 (59.3)	
concurrent chemoradiotherapy	39 (40.2)	41 (28.3)	
Immunotherapy or not			0.320
yes	2 (2.1%)	8 (5.5%)	
no	95 (97.9%)	137 (94.5%)	
If PCI after first-line treatment			0.013
yes	2 (2.1)	15 (10.3)	
no	95 (97.9)	130 (89.7)	
Extracranial progression at diagnosis of BM			0.907
yes	20 (20.6)	29 (20.0)	
no	77 (79.4)	116 (80.0)	
Radiotherapy for brain metastasis			0.733
yes	80 (82.5)	122 (84.1)	
no	17 (17.5)	23 (15.9)	

LD: Limited disease; ED: Extensive disease; PCI: Prophylactic cranial irradiation; BM: Brain metastasis; KPS: Karnofsky Performance Scale.

## Data Availability

The data that support this study are not openly available due to ethical and privacy concerns and are available from the corresponding author upon reasonable request.
